# Effectiveness of exercise intervention on muscle mass, muscle strength, and physical function among postmenopausal women with sarcopenia: a systematic review and meta-analysis

**DOI:** 10.3389/fpubh.2026.1758325

**Published:** 2026-05-20

**Authors:** Yanan Deng, Linfang Xu, Ruben Martin-Payo, Kexin Deng, Lei Huang, Yonghong Yang, Zhihao Xie, Mengying Qiu, Chuanhao Li, Fengying Zhang

**Affiliations:** 1 West China School of Nursing, Nursing Key Laboratory of Sichuan Province, Innovation Center of Nursing Research, West China Hospital, Sichuan University, Chengdu, Sichuan, China; 2 PRECAM Research Group, Health Research Institute of Asturias (ISPA), Oviedo, Spain; 3 Faculty of Medicine and Health Sciences, Universidad de Oviedo, Oviedo, Spain; 4 Department of Dermatology & Venerology, West China Hospital, Sichuan University, Chengdu, Sichuan, China; 5 Key Laboratory of Rehabilitation Medicine in Sichuan Province, West China Hospital, Sichuan University, Chengdu, Sichuan, China; 6 Rehabilitation Medicine Center and Institute of Rehabilitation Medicine, West China Hospital, Sichuan University, Chengdu, Sichuan, China; 7 School of English Studies, Sichuan International Studies University, Chongqing, China; 8 Department of Critical Care Medicine, West China Hospital, Sichuan University, Chengdu, Sichuan, China

**Keywords:** exercise intervention, muscle mass, muscle strength, physical function, postmenopausal, sarcopenia

## Abstract

**Background:**

Sarcopenia is highly prevalent among postmenopausal women due to hormonal changes and aging, and is associated with adverse health outcomes. Exercise has been proposed as a key strategy to mitigate sarcopenia; however, its effectiveness in this population remains inconclusive.

**Objective:**

To systematically evaluate the effects of different exercise modalities on muscle mass, muscle strength, and physical function in postmenopausal women with sarcopenia.

**Methods:**

A systematic search was conducted across six electronic databases from inception to April 1, 2025. Randomized controlled trials evaluating exercise interventions in postmenopausal women with sarcopenia were included. Primary outcomes were muscle mass, muscle strength, and physical performance. Meta-analyses were performed using RevMan and Stata.

**Results:**

A total of 17 RCTs involving 744 postmenopausal women with sarcopenia were included in this systematic review. The pooled analysis revealed that exercise interventions significantly improved skeletal muscle mass index. Additionally, significant improvements were found in muscle strength, including grip strength and knee extension strength. Physical function also improved, as evidenced by improvements in gait speed, the Timed Up and Go test, and single-leg stance. No significant differences were observed for body mass index (BMI) between intervention and control groups.

**Conclusions:**

Exercise interventions were associated with improvements in muscle mass, muscle strength, and physical function in postmenopausal women with sarcopenia. These findings support the integration of structured exercise programs into clinical practice to improve sarcopenia-related outcomes in this population.

**Systematic review registration:**

https://www.crd.york.ac.uk/PROSPERO/view/CRD42024608200, identifier: CRD42024608200.

## 1 Introduction

Sarcopenia is a multifaceted age-related syndrome characterized by progressive loss of skeletal muscle mass, strength, and physical function (1). It is linked to adverse outcomes such as falls, fractures, cognitive decline, and mortality, directly or indirectly increasing overall health care costs (2, 3). Notably, there are significant sex-based differences in the incidence and prevalence of sarcopenia (4). A study conducted in Korea observed a higher prevalence of sarcopenia among females (26.4%) compared to males (22.8%) (5). A longitudinal study reported an incidence of sarcopenia of 16.7% in women and 11.5% in men (6). These disparities are likely driven by underlying biological mechanisms. In postmenopausal women, the decline in estrogen levels plays a critical of accelerated muscle loss (7). Studies have shown that the incidence of sarcopenia increased from 3.8% in middle-aged women to 10.3% in postmenopausal women (8). Estrogen exerts protective effects on muscle by regulating protein synthesis and oxidative stress, whereas its decline induces a pro-inflammatory state and impairs neuromuscular function, which increases susceptibility to sarcopenia (9, 10). In addition, the impaired mitochondrial function induced by estrogen deficiency may influence responsiveness to exercise interventions. These findings underscore the need to consider sex-specific biological characteristics, particularly in postmenopausal women, when investigating the development and management of sarcopenia.

Currently, there are no widely available pharmacological treatments for managing sarcopenia, despite the substantial unmet medical demand in this domain (8). Exercise is widely recognized as the cornerstone of non-pharmacological management and has been associated with improvements in muscle mass, muscle strength, and physical performance, thereby reducing the risk and progression of sarcopenia (11). Various exercise modalities, such as resistance training, balance training, and multimodal interventions, have demonstrated beneficial effects on functional capacity and neuromuscular performance (12, 13). Nevertheless, the magnitude and consistency of these effects vary substantially across studies. This heterogeneity arises not only from differences in outcome domains but also from variability in intervention characteristics, population features, and study design factors, such as type, intensity, and duration of exercise, as well as age, baseline function, and measurement methods. Such variability limits the comparability of findings. Therefore, a systematic synthesis that explicitly accounts for these sources of heterogeneity is warranted to better clarify the effectiveness of exercise interventions and to identify optimal intervention strategies for specific populations. However, whether this heterogeneity is partly driven by sex-specific differences remains unclear.

Existing systematic reviews of exercise interventions have predominantly synthesized data from mixed-sex populations. To our knowledge, recent systematic reviews and meta-analyses have examined exercise or non-pharmacological interventions for sarcopenia management in women or older populations (14, 15). However, these studies have not specifically focused on postmenopausal women with sarcopenia as a distinct population. This distinction is critical, as postmenopausal women exhibit unique hormonal and physiological characteristics, particularly estrogen deficiency, which may influence muscle metabolism and responsiveness to exercise interventions. Consequently, synthesizing evidence specifically in this population is necessary to generate more precise and clinically relevant conclusions. Due to the marked anatomical and physiological differences between sexes that influence responses to exercise interventions (16), pooling male and female participants within the same analysis may obscure sex-specific effects (17). Differences in muscle composition, hormonal status, and baseline physical capacity may result in differential responsiveness to exercise. As a result, pooled estimates from mixed-sex populations may overestimate or underestimate the true effects in postmenopausal women, reducing the validity and applicability of the findings.

Given the current research gap, there is a pressing need to focus on postmenopausal women with sarcopenia and to develop more evidence regarding the specific effects of exercise interventions on this condition. Therefore, this systematic review and meta-analysis aimed to: (1) determine the pooled effects of exercise interventions on muscle mass, muscle strength, and physical function in postmenopausal women with sarcopenia; and (2) explore potential effect modifiers, including exercise type, duration, frequency, and diagnostic criteria, through subgroup analyses to inform more targeted and clinically relevant exercise recommendations for this population.

## 2 Method

This systematic review and meta-analysis adhered to the Preferred Reporting Items for Systematic Reviews and Meta-Analyses (PRISMA) guidelines (18) and the Cochrane Handbook for Systematic Reviews of Interventions (19). Additionally, the protocol for this systematic review and meta-analysis has been registered on the International Prospective Register of Systematic Reviews. The protocol was registered in PROSPERO (CRD42024608200).

### 2.1 Inclusion and exclusion criteria

Inclusion and exclusion criteria for this study were established based on the purpose of the study and the Population, Intervention, Comparison, Outcome, and Study design framework.

#### 2.1.1 Inclusion criteria

(1) Population: Studies involving postmenopausal women diagnosed with sarcopenia; (2) Intervention: Any exercise intervention aimed at improving sarcopenia-related outcomes, with no restrictions on the nature, form, setting, duration, or frequency; (3) Comparison: Control group that received no exercise intervention or alternative exercise intervention; (4) Study design: Randomized controlled trials; (5) Outcome: Outcomes were categorized as primary and secondary, and each study had at least one eligible outcome. The primary outcomes included: (a) muscle mass; (b) muscle strength; (c) physical function; (d) secondary outcomes. No restrictions were placed on outcome measurement methods.

#### 2.1.2 Exclusion criteria

We excluded the following studies: (a) Mixed-sex studies that did not provide separate statistical analyses; (b) Studies using qualitative data as an outcome measure; (c) Studies not written in English, and (d) Studies not using exercise intervention as an intervention for postmenopausal women.

### 2.2 Search and screen strategy

A comprehensive search was conducted across six electronic databases (PubMed, Web of Science, EMBASE, Scopus, EBSCO, and Cochrane Central) from their inception to April 1, 2025. The search strategy utilized a combination of Medical Subject Headings (MeSH) and entry terms, systematically combined using Boolean operators (“AND” and “OR”). The complete search strategy is provided in Supplementary Appendix 1. Additionally, the researchers manually searched the reference lists from included studies. The search strategy was developed and refined based on established guidelines.

### 2.3 Risk of bias assessment

The revised Cochrane Risk of Bias tool (Cochrane, London, United Kingdom) (20) was used to systematically assess the risk of bias in the included studies. Three classifications for the overall risk of bias were established: high risk, some certain, and low risk (21). Disagreements were resolved between the two reviewers through discussion.

### 2.4 Data extraction

Literature management and the identification of duplicate records were performed using EndNote 21.0 (Clarivate, Philadelphia, PA, United States). Following duplicate removal, two reviewers independently screened the titles, abstracts, and full texts of the remaining records to assess eligibility (19). For the included studies, data were independently extracted by two reviewers using a standardized form adapted from the Cochrane Collaboration. To ensure transparency, the following variables were systematically extracted: (1) study characteristics (author, year, and country); (2) participant characteristics (sample size, age, and baseline status); (3) intervention details (type, frequency, and duration); (4) control group protocols; (5) reported adverse events; and (6) outcome measures (muscle mass, muscle strength, physical function, and body composition). All extracted data were cross-checked for accuracy, and any discrepancies were resolved through discussion or consultation with a third reviewer. In cases of missing or unclear data, the original authors were contacted for clarification. Studies with missing outcome data were excluded from the corresponding meta-analysis.

### 2.5 Statistical analysis

Review Manager 5.4.1 (The Cochrane Collaboration, Copenhagen, Denmark) was used for the quantitative analysis. Stata 18.0 (StataCorp LLC, College Station, TX, United States) was used to perform Egger's regression. The standardized mean difference with 95% confidence intervals was used to pool the effects, following the Cochrane Handbook (22). The magnitude of effect sizes was interpreted according to Cohen's criteria, where 0Standardized Mean Difference (SMD) values of 0.2, 0.5, and 0.8 represent small, moderate, and large effects, respectively (23). If the required change scores were not reported, the standard deviation (SD) of the change was calculated as: [SDpre^2^ + SDpost^2^ – 2 × Corr (pre, post) × SDpre × SDpost] 0.5, with Corr (pre, post) represent the correlation, which was set to 0.5 if no correlation was reported (13, 24, 68).

Heterogeneity across studies was quantified using the *I*^2^ statistic, with 25, 50, and 75% indicating low, medium, and high heterogeneity, respectively. A random-effects model was applied when substantial heterogeneity was present (*I*^2^ > 50%), while a fixed-effect model was used otherwise (24). Subgroup analyses were conducted when the number of studies exceeded 10 or when significant heterogeneity was observed in the outcome measures (19), stratified by exercise type, intervention duration, exercise frequency, and sarcopenia diagnostic criteria. Egger's test and funnel plot were used to assess publication bias (25). Sensitivity analysis was performed by sequentially excluding studies to evaluate their impact on the aggregated effect (19, 26).

## 3 Result

### 3.1 Study selection

**Figure 1:**
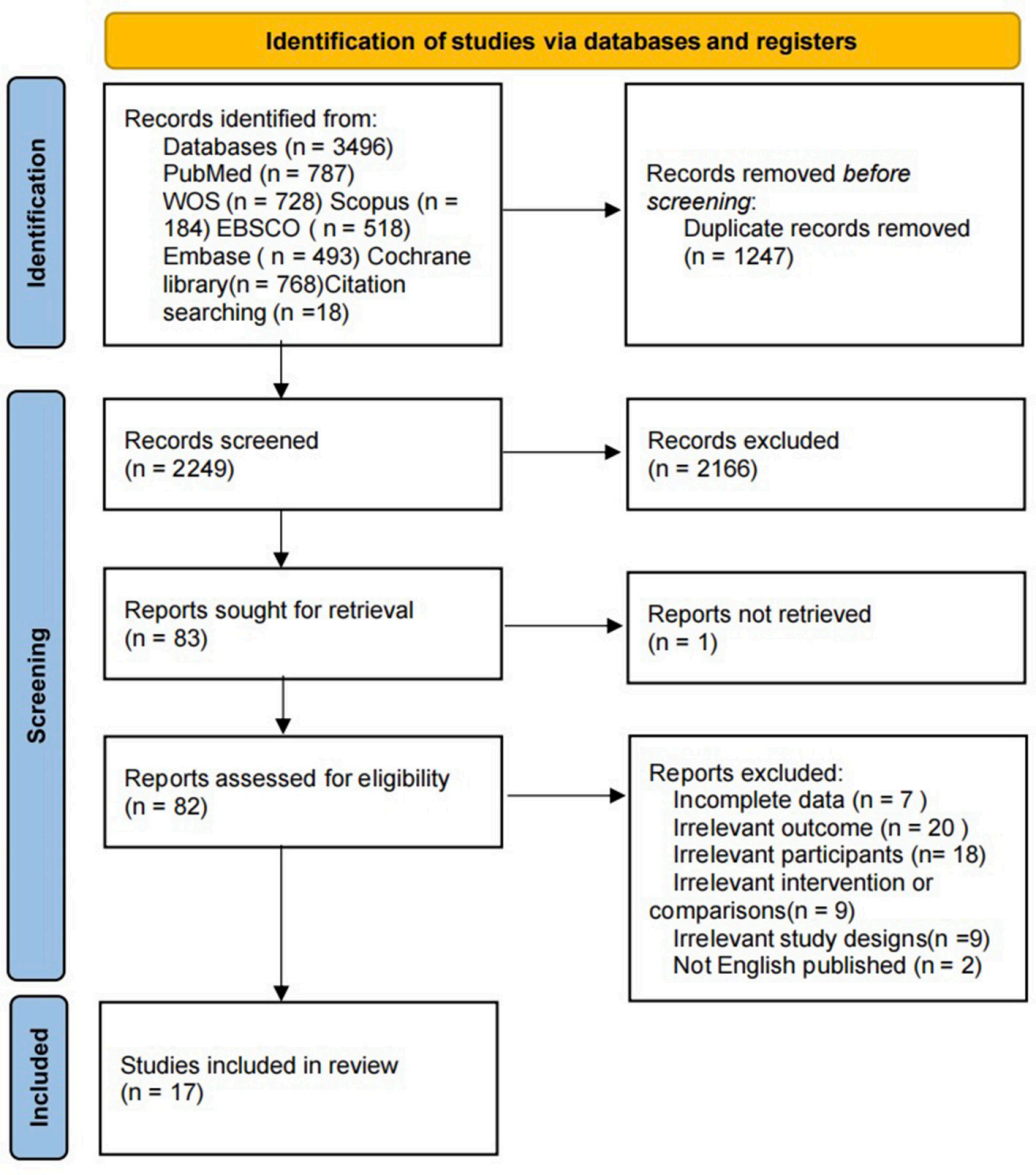
PRISMA flow diagram of data collection.

The initial database search identified 3,496 articles. A total of 2,249 were then screened based on their titles and abstracts. Additionally, three articles identified through citation searches were included in the review. Other articles were excluded due to the study design not meeting the inclusion criteria or because their full texts were non-retrievable. Finally, 17 studies involving 744 participants were included in the review. Figure 1 shows the screening process.

### 3.2 Study characteristics

A total of 17 randomized controlled trials (RCTs) published between 2016 and 2024 were included. Geographically, the evidence base is concentrated in East Asia, which accounted for 64.7% of the total studies (*n* = 11, conducted in China, Japan, and South Korea). The remaining studies were distributed across Europe (*n* = 2; Italy and Spain), the Americas (*n* = 3; United States and Brazil), and Africa (*n* = 1; Egypt). The mean age of participants ranged from 58.2 to 81.4 years. Overall participant adherence was high, ranging from 72.4 to 97.6%, although lower adherence was observed in high-intensity resistance training programs. Further details of the study characteristics are provided in Table 1.

**Table 1:** Characteristics of included studies.

Author/year/country	Sample size (E: I/F, C: I/F)	Age (E, C)	Diagnosis criteria	Adverse reactions	Outcomes
Kim H/2013/Japan	E: 30/32, C: 28/32	E: 79.6 ± 4.2, C: 80.2 ± 5.6	Study-specific criteria	NR	Muscle mass: ASM (BIA) Muscle strength: GS, KES Physical function: TUG, gait speed, SLS Body composition: BF
Kim H/2016/Japan	E: 34/35, C: 34/24	E: 81.4 ± 4.3, C: 81.1 ± 5.1	Study-specific criteria	NR	Muscle mass: SMI (BIA), ASM Muscle strength: GS, KES Physical function: gait speed Body composition: BF
Vasconcelos KS/2016/Brazil	E: 14/16, C: 14/15	E: 72.0 ± 4.6, C: 72.0 ± 3.6	Study-specific criteria	Reported with details	Muscle strength: KES Physical function: gait speed
Hamaguchi K/2017/United States	E: 6/7, C: 8/9	E: 60.4 ± 2.7, C: 60.6 ± 2.4	EWGSOP	None reported	Muscle mass: SMI Muscle strength: GS, KES
Huang SW/2017/China Taiwan	E: 17/17, C: 18/18	E:68.9 ± 4.9, C:69.5 ± 5.1	Study-specific criteria	None reported	Muscle mass: SMI (DXA) Body composition: BF, BMI
Liao CD/2017/China Taiwan	E: 23/25, C: 18/21	E: 66.4 ± 4.5, C: 68.4 ± 5.9	EWGSOP	None reported	Muscle mass: SMI (BIA) Muscle strength: GS Physical function: gait speed, TUG, TCR, SLS Body composition: BF
Chen HT/2018/China Taiwan	E: 17/17, C: 16/16	E: 66.7 ± 5.3, C: 68.3 ± 2.8	AWGS	NR	Muscle mass: SMI (DXA), ASM Muscle strength: GS Body composition: BF
Liao CD/2018/China Taiwan	E: 29/33, C: 18/23	E: 66.7 ± 4.5, C: 68.3 ± 6.1	Study-specific criteria	NR	Muscle mass: SMI (DXA) Muscle strength: GS Physical function: gait speed, SLS, TUG, TCR
Piastra G/2018/Italy	E: 35/32, C: 37/37	E: 69.9 ± 2.7, C: 70.0 ± 2.8	EWGSOP	NR	Muscle mass: SMI (BIA) Muscle strength: GS
Jung WS/2019/Korea	E: 13/13, C: 13/13	E: 75.0 ± 3.9, C: 74.9 ± 5.2	Study-specific criteria	NR	Muscle mass: ASM (DXA) Physical function: gait speed Body composition: BMI
El-Hak/2021/Egypt	E: 20/20, C: 20/20	E: 58.3 ± 2.8, C: 58.2 ± 3.1	EWGSOP	NR	Muscle mass:muscle mass (%; BIA) Muscle strength: GS Physical function: gait speed, TCR
Lee YH/2021/China Taiwan	E: 15/15, C: 12/12	E: 70.1 ± 4.1, C: 71.8 ± 5.2	EWGSOP	None reported	Muscle mass: SMI (DXA) Muscle strength: GS Physical function: gait speed, SLS, TUG, TCR Body composition: BF
Seo MW/2021/Korea	E: 12/14, C: 10/13	E: 70.3 ± 5.4, C: 72.9 ± 4.8	IWGS, EWGSOP	NR	Muscle mass: ASM (DXA) Muscle strength: GS Physical function: gait speed, TUG, SLS, TCR Body composition: BMI
Chen BY/2023/Korea	E: 25/30, C: 24/30	E: 65.7 ± 2.5, C: 65.2 ± 2.6	AWGS	None reported	Muscle mass: SMI, ASM (BIA) Muscle strength: GS Physical function: gait speed, TUG
Flor-Rufino C/2023/Spain	E: 20/27, C: 18/24	E: 79.9 ± 7.2, C: 79.6 ± 7.7	EWGSOP	Reported with details	Muscle mass: SMI (BIA, MRI) Muscle strength: GS Physical function: gait speed, TUG
Valdés-Badilla P/2023/United States	E: 21/22, C: 19/22	E: 73.9 ± 8.3, C: 72.9 ± 8.7	EWGSOP	None reported	Muscle strength: GS, KES Physical function: gait speed, TUG
Jung WS/2024/Korea	E: 14/14, C: 14/14	E: 78.1 ± 3.7, C: 78.2 ± 3.7	Study-specific criteria	NR	Muscle mass: ASM (DXA) Body composition: BF, BMI

E, exercise group; C, control group; I, initial intervention number; F, final intervention number; EWGSOP, European Working Group on sarcopenia in older people; AWGS, Asian Working Group for sarcopenia; IWGS, International Working Group on sarcopenia; NA, not applicable; NR, not reported; DXA, dual-energy X-ray absorptiometry; BIA, bioelectrical impedance analysis; ASM, appendicular skeletal muscle mass; BMI, body mass index; SMI, skeletal muscle mass index; BF, body fat; KES, knee extension strength; SLS, single leg stance; GS, grip strength; TUG, timed up and go; TCR, timed chair rise.

### 3.3 Exercise and control interventions

A wide range of exercise modalities was employed across the included trials, including power training [kettlebell training (27), power training (28–30), elastic resistance training (31–36), and mixed training (37–42, 66), and Chen (43)]. Control group management across the included trials was heterogeneous and could be broadly categorized into passive and active control conditions. Passive controls included maintenance of usual activities, health education programs, and telephone-based monitoring. In contrast, active control conditions involved alternative exercise interventions, such as dance (36), postural training (29), and walking programs (41). This variability in control conditions may influence the magnitude of between-group differences and should be considered when interpreting the pooled results. Table 2 summarizes the characteristics of the included studies.

**Table 2:** Characteristics of the exercise interventions in included studies.

Type	References	Exercise interventions				Control group
		Content	Duration, frequency, session	Intensity	Venue	
Elastic resistance training	Huang et al. (32)	Elastic band resistance training	12 weeks, 3 days/week, 55 min	Progressive	Academic medical center	Health education
	Liao et al. (33)	Elastic band resistance training	12 weeks, 3 days/week, 45–55 min	Moderate intensity progressive	Hospital	Usual activity
	Liao et al. (34)	Elastic band resistance training	12 weeks, 3 days/week, 55 min	Moderate intensity progressive (Borg)	Hospital	Usual activity
	Lee et al. (35)	Elastic band resistance training	12 weeks, 3 days/week, 55 min	Progressive (Borg)	Hospital	Health education
	Seo et al. (36)	Elastic band resistance training	16 weeks, 3 days/week, 60 min	Progressive (OMNI)	NR	Usual activity
	Valdés-Badilla et al. (37)	Elastic band resistance training	12 week, 3 days/week, 60 min	Moderate to vigorous	Laboratory	Active control (group-based dance)
Mixed training	Kim et al. (38)	Stretching, strengthening, balance, gait training	12 weeks, 2 days/week, 60 min	Moderate intensity progressive	Institute	Health education
	Hamaguchi et al. (40)	Circuit resistance training, stretching and cycling exercises	6 weeks, 2 days/week, 60 min	Progressive	University	Usual activity
	Jung et al. (41)	Circuit training	12 weeks, 3 days/week, 25–55 min	Phase adjustability (HRR)	NR	Usual activity
	El-Hak et al. (66)	Core Exercises, low caloric diet; walking	12 weeks, 3 days/week, 30 min	NR	Hospital	Active control (low caloric diet, walking)
	Chen et al. (43)	Tai Chi and progressive resistance training	8 weeks, 3 days/week, 60 min	Moderate intensity progressive (Borg)	Activity room	Health education
	Jung et al. (42)	Circuit training	12 weeks, 3 days/week, 45–75 min	Moderate intensity (HRR)	NR	Usual activity
Power training	Kim et al. (39)	Resistance band training; weight-bearing training; aerobic training	12 weeks, 2 days/week, 60 min	Progressive individual adaptation	Institute	Health education
	Flor-Rufino et al. (31)	High-intensity circuit resistance training (HIRT)	24 weeks, 2 days/week, 65 min	High-intensity (OMNI)	University	Telephone follow-up
	Vasconcelos et al. (29)	Open and closed chain training	10 weeks, 2 days/week, 60 min	Progressive	University	Telephone follow-up
	Piastra et al. (30)	Low-load muscle strengthening	36 weeks, 2 days/week, 60 min	Low to moderate, progressive	Gym	Active control (posture training)
	Chen et al. (28)	Kettlebell training	8 weeks, 2 days/week, 60 min	Progressive (RPE)	Hospital	Usual activity

RPE, rating of perceived exertion; OMNI, OMNI resistance active muscle inventory; HRR, heart rate reserve; Borg, Brog self-perceived exertion scale; NR, not reported; Missing or unreported information (NR) was not imputed and was excluded from relevant subgroup analyses where applicable.

### 3.4 Risk of bias assessment

**Figure 2:**
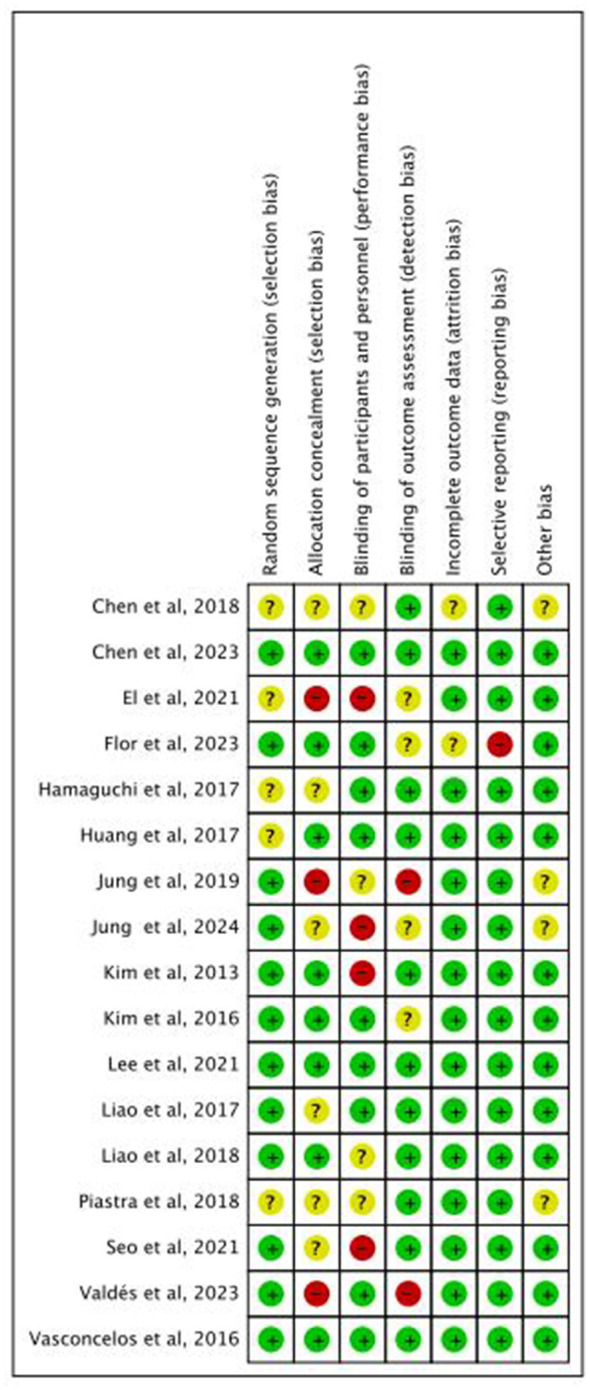
Risk of bias assessment.

Figure 2 summarizes the methodological risk of bias assessment of 17 studies. During quality assessment, independent reviewers initially reached 94% consensus. Any disagreements that arose were addressed through discussions, ultimately resulting in 100% agreement on bias classification.

In the review, twelve reported randomized sequence generation (low selection bias risk), while three had inadequate allocation concealment (high selection bias risk). Blinding of participants was unfeasible due to the nature of exercise intervention, with eight studies showing high or unclear bias risk in this domain. Two studies did not blind outcome assessors, and another two lacked sufficient outcome data completeness information. One study had high risk of other biases, and 13 had low risk. This assessment highlighted the variability in methodological quality across the included trials. Sensitivity analyses were conducted to assess the influence of high-risk studies on pooled estimates.

### 3.5 Synthesis of results

#### 3.5.1 Effectiveness of exercise intervention on the muscle mass

**Figure 3:**
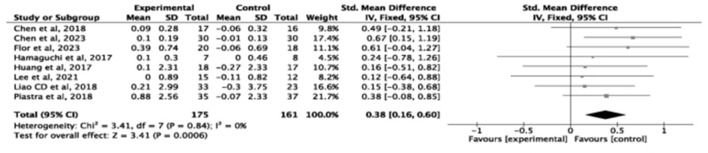
Forest plot of the effect of exercise interventions on skeletal muscle mass index.

**Figure 4:**
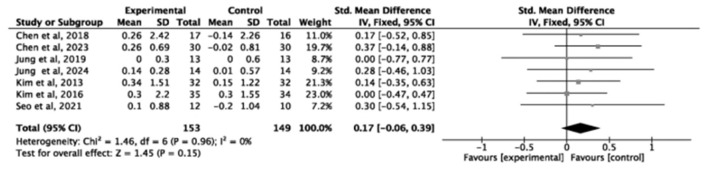
Forest plot of the effect of exercise interventions on appendicular skeletal muscle index.

A total of seven studies (*n* = 336) assessed skeletal muscle mass index (SMI). Exercise significantly improved the skeletal muscle mass index compared with the control group (SMD = 0.38, small effect, 95% *CI* = 0.16–0.60, *P* = 0.0006, *I*^2^ = 0%; Figure 3). In contrast, eight studies (*n* = 302) evaluated appendicular skeletal muscle index showed no significant effect (SMD = 0.17, 95% *CI* = −0.06 to 0.39, *P* = 0.15, *I*^2^ = 0%; Figure 4).

#### 3.5.2 Effectiveness of exercise intervention on muscle strength

**Figure 5:**
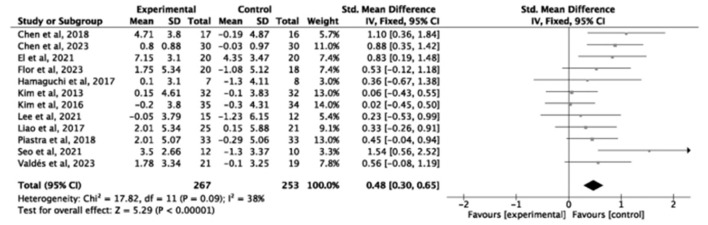
Forest plot of the effect of exercise interventions on grip strength.

**Figure 6:**
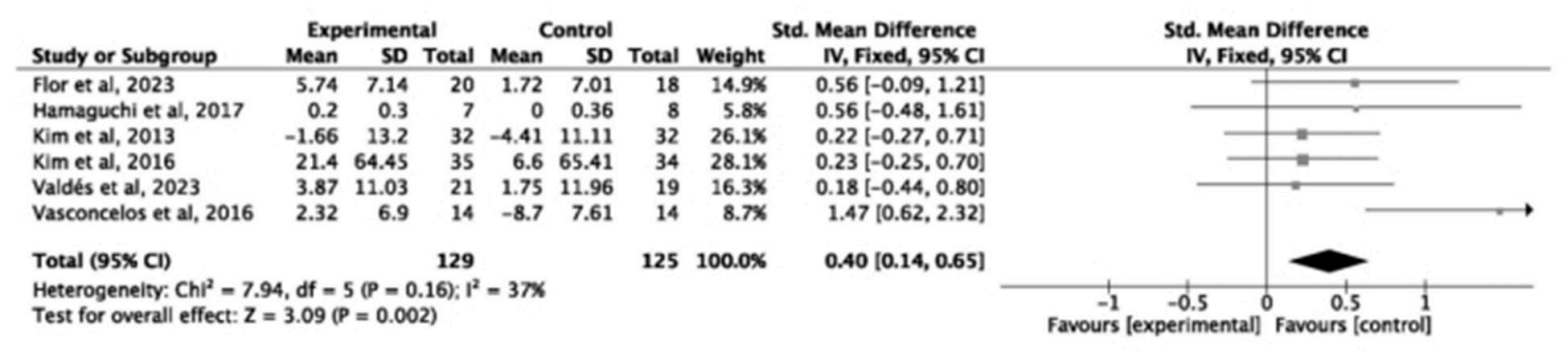
Forest plot of the effect of exercise interventions on knee extension strength.

Twelve studies (*n* = 520) reported grip strength, demonstrating a significant improvement following exercise intervention (SMD = 0.48, small-to-moderate effect 95% *CI* = 0.30–0.65, *P* < 0.00001, *I*^2^ = 38%; Figure 5). Similarly, six studies (*n* = 254) showed enhanced knee extension strength (SMD = 0.40, small-to-moderate effect, 95% *CI* = 0.14–0.65, *P* = 0.002, *I*^2^ = 37%; Figure 6), indicating consistent strength gains.

#### 3.5.3 Effectiveness of exercise intervention on physical function

**Figure 7:**
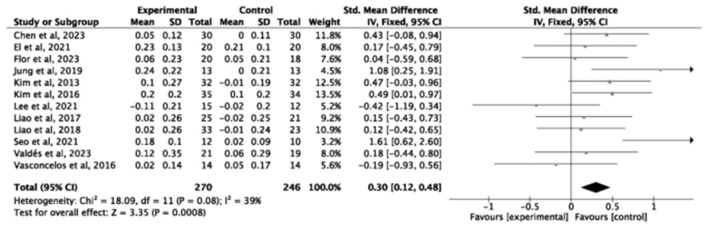
Forest plot of the effect of exercise interventions on gait speed.

**Figure 8:**
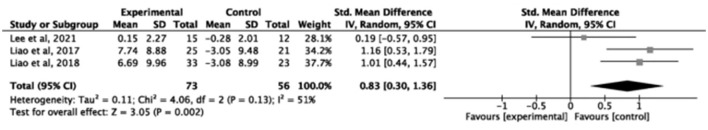
Forest plot of the effect of exercise interventions on single-leg stance.

**Figure 9:**
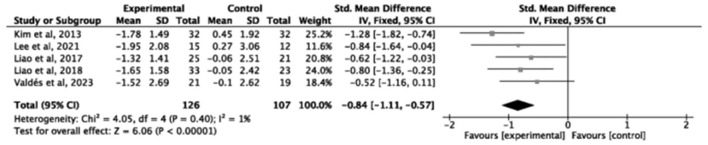
Forest plot of the effect of exercise interventions on TUG.

**Figure 10:**
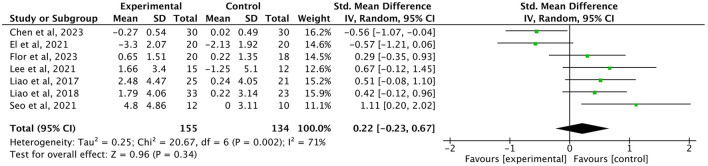
Forest plot of the effect of exercise interventions on timed chair rise.

Gait speed (GS) was assessed in 12 studies (*n* = 516), showing a significant improvement (SMD = 0.30, small effect, 95% *CI* = 0.12–0.48, *P* = 0.0008, *I*^2^ = 38%; Figure 7). Three studies (*n* = 129) examined single-leg stance, (SMD = 0.83, large effect, 95% *CI* = 0.30–1.36, *I*^2^ = 51%, *P* = 0.002; Figure 8). Five studies (*n* = 233) evaluating the Time Up and Go test (TUG; SMD = −0.84, large effect, 95% *CI* = −1.11 to −0.57, *P* < 0.00001, *I*^2^ = 1%; Figure 9). In contrast, no significant difference was found for timed chair rise across seven studies (*n* = 134, SMD = 0.22, 95% *CI* = −0.23 to 0.67, *P* = 0.34, *I*^2^ = 71%; Figure 10).

#### 3.5.4 Effectiveness of exercise intervention on body fat, BMI

Six studies involving a total of 247 participants were analyzed, and the results indicated a modest but significant reduction in body fat between the exercise intervention group and the control group (SMD = −0.27, small effect, 95% *CI* = −0.52 to −0.02). Five studies with 151 participants also found no significant differences in BMI reduction between those undergoing exercise intervention and the control group (SMD = 0.08, 95% *CI* = – 0.40 to 0.24; see Supplementary Appendix 1).

### 3.6 Subgroup analyses

Based on the characteristics of the included studies, subgroup analyses were performed to examine the effects of different exercise intervention programs on muscle mass, muscle strength, and physical function. These analyses focused on different exercise types, durations, frequencies, and sarcopenia diagnostic criteria.

Elastic band training demonstrated a significant effect on grip strength (SMD = 0.51, 95% *CI* = 0.10–1.04, *P* = 0.02, *I*^2^ = 42%, *n* = 135), outperforming mixed training; 3-weekly training (SMD = 0.57, moderate effect, 95% *CI* = 0.37–0.99, *P* < 0.0001, *I*^2^ = 25%, *n* = 235) showed greater efficacy than twice-weekly training. The intervention effect on grip strength was most pronounced when the intervention duration was < 12 weeks (SMD = 0.87, large effect, 95% *CI* = 0.47–1.27, *P* < 0.0001, *I*^2^ = 0%, *n* = 108); when the European Working Group on Sarcopenia in Older People criteria were used to diagnose sarcopenia, the intervention had the best effect on grip strength (SMD = 0.54, moderate effect, 95% *CI* = 0.31 to 0.78, *P* < 0.00001, *I*^2^ = 0%, *n* = 93).

For physical function, both elastic band training and mixed training significantly improved timed chair rise, with elastic band training (SMD = 0.58, moderate effect, 95% *CI* = 0.25 to 0.91, *P* = 0.0006, *I*^2^ = 0%, *n* =151) demonstrating superior efficacy compared to mixed training. However, the overall effect on timed chair rise was not statistically significant (*I*^2^ = 76%), likely attributable to heterogeneity in measurement methods across studies. Improvement in gait speed was most pronounced at the 12-week intervention period (SMD = 0.18, small effect, 95% *CI* = 0.05–0.52, *P* = 0.02, *I*^2^ = 21%, *n* = 368). With elastic band training (SMD = 0.49, small-to-moderate effect, 95% *CI* = 0.09–0.88, *P* = 0.02, *I*^2^ = 61%, *n* = 271) and the Others diagnostic criteria subgroup (SMD = 0.41, small-to-moderate effect, 95% *CI* = 0.15–0.58, *P* = 0.003, *I*^2^ = 7%, *n* = 243) showing the most pronounced effects.

### 3.7 Publication bias

Funnel plots were employed to assess publication bias in this meta-analysis. Among the evaluated metrics, potential publication bias could have been present for both the assessments of grip strength and gait speed. More details can be found in Supplementary Appendix 1. Additional analyses using Egger's regression test were conducted to further evaluate publication bias. The results for grip strength (*t* = 0.50, *P* = 0.6302) and gait speed (*t* = 1.69, *P* = 0.1222) both indicated no statistically significant differences. These findings suggest that there is no evidence of publication bias for either metric, supporting the stability of the meta-analysis results. Although not statistically significant, potential publication bias cannot be excluded.

### 3.8 Sensitivity analyses

Sensitivity analyses were conducted to assess the robustness of the pooled results by excluding studies with a high risk of bias in key domains, particularly allocation concealment and blinding of outcome assessment (*n* = 3). After exclusion, the pooled effect sizes for the primary outcomes remained largely unchanged, with statistically significant effects observed for grip strength (SMD = 0.44, 95% *CI* = 0.25–0.63), and gait speed (SMD = 0.28, 95% *CI* = 0.08–0.48). These findings were consistent with the original estimates, indicating that studies at high risk of bias did not materially influence the overall results and supporting the robustness of the meta-analysis.

## 4 Discussion

This systematic review and meta-analysis, focusing exclusively on postmenopausal women with sarcopenia, provides evidence suggesting that exercise interventions may be beneficial across multiple domains of sarcopenia. The pooled analyses indicate that exercise significantly improved muscle strength and physical function, whereas the effects on muscle mass were less consistent. The following sections discuss these findings in the context of existing evidence and their implications for clinical practice.

### 4.1 Muscle mass

This study demonstrates that exercise interventions were associated with improvements in skeletal muscle mass index (SMI) in postmenopausal women with sarcopenia, supporting the important role of physical activity in managing this condition. These findings are consistent with previous meta-analyses, such as Zhao (44), which reported that resistance exercise has been associated with improvements in muscle mass in older adults with sarcopenia. However, no significant effect was observed for appendicular skeletal muscle mass index (ASM), as reported in previous studies (45), who also reported non-significant effects of exercise interventions on appendicular muscle mass. One possible explanation is that SMI reflects overall muscle status, whereas ASM specifically captures limb muscle mass, which may respond differently to exercise stimuli. In addition to physiological differences, methodological differences in muscle mass assessment may also have contributed to the inconsistent findings. Among the included studies, nine used bioelectrical impedance analysis (BIA) and seven used dual-energy X-ray absorptiometry (DXA). BIA is sensitive to hydration status and may underestimate or overestimate changes in muscle mass (46), whereas DXA is generally considered a more precise and reliable method for body composition assessment (47, 48). This heterogeneity in measurement techniques may have influenced effect size estimation and contributed to the divergence in results across studies.

### 4.2 Muscle strength

Exercise interventions were associated with significant improvements in grip strength and knee extension strength. These findings align with those of Zhao (44) and Wu (49). Furthermore, subgroup analysis suggested that training performed three times weekly appeared to be associated with greater improvements on grip strength compared with twice-weekly sessions. The observation may be related to the increased synthesis of myofibrillar proteins and metabolic signals (50) alongside enhanced activation of motor units (51), all of which may contribute to improved grip strength. Further analyses indicated that elastic band training was associated with improvements in grip strength, consistent with findings reported by Hernandez (52). The varying resistance provided by elastic bands may stimulate neuromuscular activation, contributing to grip strength gains. However, because of the limited number of studies within each modality subgroup and the potential for multiple comparisons, these between-modality differences should be interpreted with caution and considered exploratory in nature. Discrepancies in results across studies may reflect individual variations in physical function, muscle mass, baseline health, and neuromuscular control among postmenopausal women with sarcopenia.

### 4.3 Physical function

Exercise interventions were associated with significant improvements in gait speed, single-leg stance, and TUG, consistent with previous findings (53–55). Contrary to the results of Cheng (56), these discrepancies may reflect variations in study populations, as their research focused on individuals with secondary sarcopenia. Subgroup analyses suggested a potential trend in which the intervention effect on GS appeared more pronounced at a duration of 12 weeks. Studies with shorter intervention durations (6–8 weeks) may provide insufficient cumulative stimulus to induce substantial neuromuscular adaptations, while longer interventions (16–24 weeks) may reflect gradual physiological adaptation of participants to exercise intensity and patterns (57, 58). This adaptation may attenuate the rate of subsequent improvement rather than reduce overall benefits. These findings suggest a potential distinction between short-term efficacy and longer-term adaptive responses to exercise interventions. However, these findings should be interpreted with caution as exploratory findings, given the limited number of studies and the potential for inflated Type I error due to multiple comparisons.

For the timed chair rise test, no overall significant effect was observed. This lack of significance may be due to discrepancies in the units of measurement across studies. Elastic band training studies reported improvements whereas mixed training studies showed declines, leading to a non-significant pooled effect. Results indicate that exercise interventions significantly improved TUG performance, which is consistent with those of multiple systematic reviews (52, 58). Improvements in TUG performance may contribute to a reduced risk of falls and enhanced overall physical function (59, 60).

### 4.4 Secondary outcomes

Exercise interventions a reduction in body fat. This finding aligns with Hsu (2) and Chen (61), who reported that exercise intervention can significantly improve body fat levels in sarcopenic older adults. However, it did not improve BMI, which aligns with Yin's research (62). It is possible that these findings may reflect reductions in fat mass alongside increases in muscle mass, resulting in a smaller change in total body weight, resulting in a non-significant change in BMI despite reduction in body fat.

### 4.5 Critique of included studies

The methodological aspects of the included studies highlight several critical issues. One primary concern is the reliance on BIA to measure muscle mass in nine studies. BIA measurements are susceptible to hydration status and are generally less accurate and reliable than DXA (63). This measurement variability may have influenced the estimation of muscle mass changes and contributed to the inconsistent findings observed across studies. Future research should prioritize the use of standardized and more precise assessment methods, such as DXA or MRI, or consider combining multiple techniques to improve measurement validity (64). Another issue is the inadequacy of follow-up duration across the studies. Only three studies assessed whether muscle and physical function benefits persisted after exercise cessation (28, 34, 64). The lack of long-term follow-up limits the ability to evaluate the durability of intervention effects and their implications for long-term health outcomes (65, 67). Additionally, reporting of adverse events and intervention details was generally inadequate. Insufficient reporting may compromise the reproducibility of interventions and obscure potential safety concerns, thereby limiting the translation of findings into clinical practice. Furthermore, substantial heterogeneity in control group conditions was a significant factor that may have influenced the estimated intervention effects. The included trials utilized both passive controls and active controls. Such variability in control group intensity can moderate the observed contrast between the intervention and control groups. In trials employing active controls, the effect of the exercise intervention might have been underestimated due to the potential benefits derived from the alternative activities in the control group. Taken together, these methodological issues highlight the need for future studies to adopt standardized outcome measurements, clearly defined control conditions, comprehensive reporting of intervention protocols and adverse events, and extended follow-up periods to enhance the robustness and comparability of the evidence.

## 5 Limitations

While the pooled analyses suggest that exercise interventions may improve muscle strength and physical function in postmenopausal women with sarcopenia, several limitations warrant consideration. First, substantial clinical and methodological heterogeneity existed among the included studies, driven by variations in diagnostic criteria and exercise protocols. The impact of this heterogeneity was outcome-specific; for instance, higher heterogeneity in the timed chair rise test (*I*^2^ = 71%) may have contributed to the non-significant pooled effect. Although a random-effects model and subgroup analyses were applied, residual heterogeneity may still have influenced the estimated effects. Second, the absence of subgroup analyses for key variables such as race, diagnostic thresholds, and exercise intensity may limit the interpretation of findings. The predominance of Asian populations may restrict generalizability, and the lack of intensity-based analyses precludes exploration of dose–response relationships. Third, although publication bias was assessed using funnel plots and Egger's test, the relatively small number of included studies may have limited the statistical power to detect such bias, and the possibility of selective reporting cannot be excluded. Finally, the evidence base was restricted to a limited range of exercise modalities, with insufficient data on emerging interventions such as whole-body vibration or blood flow restriction training, highlighting the need for future trials exploring a broader spectrum of exercise strategies in this population.

## 6 Implications for future practice and research

This systematic review identified a consistent pattern in the effects of exercise interventions on sarcopenia in postmenopausal women. Improvements in muscle strength and physical performance were more consistent and pronounced across studies, whereas changes in muscle mass were less consistent and often modest. This discrepancy may reflect differences in measurement methods, intervention duration, and underlying physiological mechanisms. Based on these findings, exercise interventions for postmenopausal women should enhance muscle strength and functional performance, such as progressive resistance training. Given the sex-specific characteristics, tailored exercise programs are needed, incorporating gradual progression in training intensity, with appropriate consideration of hormonal and physiological factors. Resistance training with lower initial loads and higher repetitions may be particularly suitable for this population. In addition, long-term follow-up studies are needed to evaluate the sustainability of intervention effects after the intervention, particularly with regard to muscle mass changes. This study may helps to address existing evidence gaps in underrepresented populations and may inform the development of more targeted and equitable exercise strategies for postmenopausal women at risk of sarcopenia.

## 7 Conclusion

This systematic review suggests that exercise interventions are associated with improvements in muscle mass, muscle strength, and physical function in postmenopausal women with sarcopenia. Given the observed sex-specific responses, tailored exercise programs for postmenopausal women are warranted.

Current evidence is limited to specific exercise modalities, with insufficient data on emerging interventions such as whole-body vibration or blood flow restriction training. Future research should explore a broader range of exercise therapies through large-scale, multicenter trials and investigate the underlying physiological mechanisms to inform the development of effective non-pharmacological interventions for this vulnerable population.

## Data Availability

Publicly available datasets were analyzed in this study. This data can be found here: the datasets analyzed in this study are derived from previously published articles, which are cited in the reference list of the manuscript. No specific repository or accession number applies.
