# Supplementary material for: Effectiveness of exercise intervention on muscle mass, muscle strength, and physical function among postmenopausal women with sarcopenia: a systematic review and meta-analysis
Source: Front Public Health. 2026 May 20;14:1758325. doi: 10.3389/fpubh.2026.1758325 (PMC13229780; doi:10.3389/fpubh.2026.1758325)
Supplement: Supplementary file 1 [file Data_Sheet_1.pdf]

Supplementary Material

Appendix 1 Search strategies....1

Table 1 Pubmed 2

Table 2 Web of Science.... 2

Table 3 SCOPUS.... 3

Table 4 EBSCO....3

Table 5 Embase....4

Table 6 Cochrane Library.... 4

Appendix 2 Meta analysis....5

2.1 meta analyses- Body Fat.... 5

2.2 meta analyses - BMI.... 5

2.3 meta analyses - Quality of Life Outcomes....5

2.4 meta analyses - Hs-CRP.... 5

Appendix 3 Subgroup analyse.... 6

3.1 Subgroup analyses-Type of exercise.... 6

3.2 Subgroup analyses - Frequency.... .7

3.2 Subgroup analyses - Duration of time.... 8

3.2 Subgroup analyses - Sarcopenia diagnostic criteria.... .9

Appendix 4 Funnel plot.... 11

Appendix 5 PRISMA checklist.... 12

Appendix 6 Detailed Description of Interventions....17

Appendix 1 Search strategies

Method

Table 1 Search strategy of PubMed	
**Number**	**Query**
#1	Mesh: Sarcopenia, Sarcopenias
#2	"Sarcopenias"[Title/Abstract] OR"Sarcopenias"[MeSH Terms] OR "Muscle wasting"[Title/Abstract] OR "Muscle atrophy"[Title/Abstract] OR "Lean body mass"[Title/Abstract] OR "Muscle mass"[Title/Abstract] OR "Muscle strength"[Title/Abstract] OR "Handgrip"[Title/Abstract]
#3	"Postmenopausal women"[Title/Abstract] OR "Midlife women"[Title/Abstract] OR "Middle aged women"[Title/Abstract] OR "Perimenopause"[Title/Abstract] OR "Menopausal transition"[Title/Abstract] OR "Climacteric"[Title/Abstract] OR OR "Women in Later Life"[Title/Abstract] OR "Aged Women"[Title/Abstract] OR "Geriatric Women"[Title/Abstract]
#4	"Exercise therapy"[Title/Abstract] OR "Remedial Exercise"[Title/Abstract] OR "Rehabilitation Exercises"[Title/Abstract] OR "Physical activities"[Title/Abstract] OR "Aerobic Exercise"[Title/Abstract] OR "Exercise Training"[Title/Abstract] OR "Exercise Intervention"[Title/Abstract] OR" balance training" OR " Resistance Training"[Title/Abstract]
#4	(#1 OR #2) AND #3 AND #4 = 630

Table 2 Search strategy of Web of Science	
Number	Query
#1	TS=("Sarcopenias" OR"Sarcopenias" OR "Muscle wasting" OR "Muscle atrophy" OR "Lean body mass" OR "Muscle mass"\ OR "Muscle strength" OR "Handgrip"
#2	TS=("Postmenopausal women"OR "Midlife women" OR "Middle aged women" OR "Perimenopause" OR "Menopausal transition" OR "Climacteric" OR "Women in Later Life" OR "Aged Women" OR "Geriatric Women")
#3	TS=("Exercise therapy"OR "Remedial Exercise"OR "Rehabilitation Exercises" OR "Physical activities" OR "Aerobic Exercise" OR "Exercise Training"OR "Exercise Intervention"OR "balance training OR "Resistance Training ")
#4	#1 AND #2 AND #3=648

Table 3 Search strategy of Scopus	
Number	Query
#1	TITLE-ABS-KEY("sarcopenia"OR"Sarcopenias" OR "Muscle wasting" OR "Muscle atrophy" OR" Lean body mass" OR "Muscle mass" OR "Muscle strength" OR "Handgrip")
#2	TITLE-ABS-KEY("Postmenopausal women" OR "Midlife women"OR "Middle aged women"OR "Perimenopause" OR "Menopausal transition" OR "Climacteric")
#3	TITLE-ABS-KEY("exercise therapy" OR "physical exercise" OR "physical activity" OR "exercise training" OR "aerobic exercise" OR "resistance exercise" OR "strength training" OR "balance exercise" OR "functional exercise")
#4	#1 AND #2 AND #3=403

Table 4 Search strategy of EBSCO	
Number	Query
#1	TI("sarcopenia") OR AB("sarcopenia") OR TI("muscle atrophy") AND AB("muscle atrophy") OR TI("aging") AND AB("aging") OR TI("age-related muscle loss") OR AB("age-related muscle loss") OR (AB("frailty") AND (TI("frailty")OR (AB("frailty") AND AB("muscle"))
#2	TI("postmenopausal women") OR AB("postmenopausal women") OR TI("post menopause women") OR AB("post menopause women") OR TI("women after menopause") OR AB("women after menopause") OR ((TI(menopause) AND TI(women)) OR (AB(menopause) AND AB(women))
#3	TI("exercise therapy") OR AB("exercise therapy") OR TI("physical exercise") OR AB("physical exercise") OR TI("physical activity") OR AB("physical activity") OR TI("exercise training") OR AB("exercise training") OR TI("aerobic exercise") OR AB("aerobic exercise") OR TI("resistance exercise") OR AB("resistance exercise") OR TI("strength training") OR AB("strength training") OR TI("balance exercise") OR AB("balance exercise") OR TI("functional exercise") OR AB("functional exercise")
#5	#1 AND #2 AND #3=148

Table 5 Search strategy of Embase	
Number	Query
#1	(Sarcopenias* OR"Sarcopenias* OR "Sarcopenias* OR Muscle atrophy* OR "Lean body mass* OR Muscle mass* OR Muscle strength* OR "Handgrip*).mp.
#2	(Postmenopausal women* or midlife women* or middle aged women* or perimenopause* or menopausal transition* or climacteric*).mp.
#3	(exercise therapy* or remedial exercise* orrehabilitation exercises* or physical activities* or aerobic exercise* or exercise training* or exercise intervention*).mp.
#4	#1 AND #2 AND #3 =453

Table 6 Search strategy of Cochrane library	
**Number**	**Query**
#1	("Sarcopenias"OR"Sarcopenias"OR "Sarcopenias" OR "Muscle atrophy" OR "Lean body mass" OR "Muscle mass" of "Muscle strength" OR "Handgrip")ti,ab,kw
#2	("Postmenopausal women"OR "Midlife women" OR "Middle aged women" OR "Perimenopause" OR "Menopausal transition" OR "Climacteric" OR "Women in Later Life" OR "Aged Women" OR "Geriatric Women")ti,ab,kw
#3	("Exercise therapy"OR "Remedial Exercise"OR "Rehabilitation Exercises" OR "Physical activities" OR "Aerobic Exercise" OR "Exercise Training" OR "Exercise Intervention"OR "balance training OR "Resistance Training ")ti,ab,kw
#4	#1 AND #2 AND #3 =751

Appendix 2 Meta analysis

Result

2.1. Body Fat

	Experimental			Control				Std. Mean Difference
Study or Subgroup	Mean	SD	Total	Mean	SD	Total	Weight	IV, Fixed, 95% CI
Chen et al, 2018	−0.79	7.69	17	0.42	6.36	16	13.7%	−0.17 [−0.85, 0.52]
Huang et al, 2017	−0.25	8.69	18	−0.1	814	17	14.6%	−0.00 [−0.66, 0.66]
Jung et al, 2024	−1.79	4.16	14	1.58	3.13	14	10.5%	−0.89 [−1.67, −0.11]
Lee et al, 2021	−2.69	7.47	15	−0.96	7.57	12	11.0%	−0.22 [−0.99, 0.54]
Liao CD et al, 2018	−0.76	3.9	33	0.68	5.1	23	22.3%	−0.32 [−0.86, 0.22]
Liao et al, 2017	−0.76	3.9	25	0.68	5.1	21	18.8%	−0.32 [−0.90, 0.27]
Seo et al, 2021	0	3.78	12	−0.1	5.1	10	9.1%	0.02 [−0.82, 0.86]
**Total (95% CI)**			**134**			**113**	**100.0%**	**−0.27 [−0.52, −0.02]**

Heterogeneity: $Chi^2 = 3.66$, df = 6 (P = 0.72); $I^2 = 0\%$
Test for overall effect: Z = 2.09 (P = 0.04)

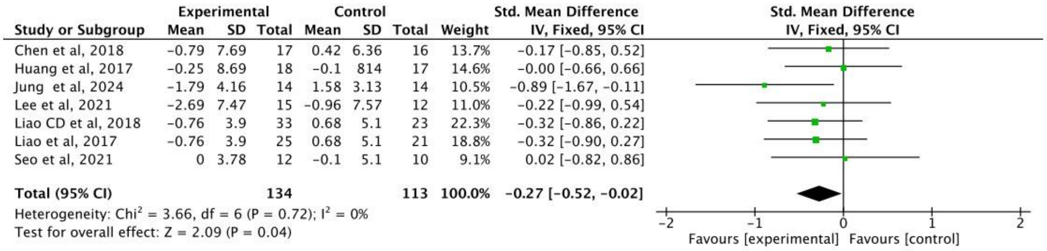

Fig.a. Forest plot of Body fat

2.2.BMI

	Experimental			Control				Std. Mean Difference
Study or Subgroup	Mean	SD	Total	Mean	SD	Total	Weight	IV, Fixed, 95% CI
El et al, 2021	−2.8	4.14	20	−2.82	4.18	20	26.6%	0.00 [−0.62, 0.62]
Huang et al, 2017	−0.05	3.9	18	−0.1	3.33	17	23.3%	0.01 [−0.65, 0.68]
Jung et al, 2019	−0.3	1.31	13	0	1.25	13	17.2%	−0.23 [−1.00, 0.54]
Jung et al, 2024	−0.3	1.8	14	0.14	1.99	14	18.5%	−0.23 [−0.97, 0.52]
Seo et al, 2021	0	1.55	12	0	2.02	10	14.5%	0.00 [−0.84, 0.84]
**Total (95% CI)**			**77**			**74**	**100.0%**	**−0.08 [−0.40, 0.24]**

Heterogeneity: $Chi^2 = 0.47$, df = 4 (P = 0.98); $I^2 = 0\%$
Test for overall effect: Z = 0.47 (P = 0.64)

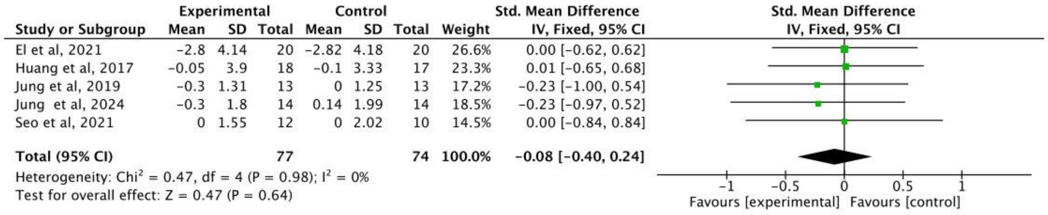

Fig.b. Forest plot of BMI

2.3. Quality of Life Outcomes

	Experimental			Control				Mean Difference
Study or Subgroup	Mean	SD	Total	Mean	SD	Total	Weight	IV, Fixed, 95% CI
Hamaguchi K et al, 2017	−1	22.07	14	6	22.52	14	19.4%	−7.00 [−23.52, 9.52]
Liao CD et al, 2018	−2.32	18.44	33	3.03	12.46	23	80.6%	−5.35 [−13.44, 2.74]
**Total (95% CI)**			**47**			**37**	**100.0%**	**−5.67 [−12.94, 1.60]**

Heterogeneity: $Chi^2 = 0.03$, df = 1 (P = 0.86); $I^2 = 0\%$
Test for overall effect: Z = 1.53 (P = 0.13)

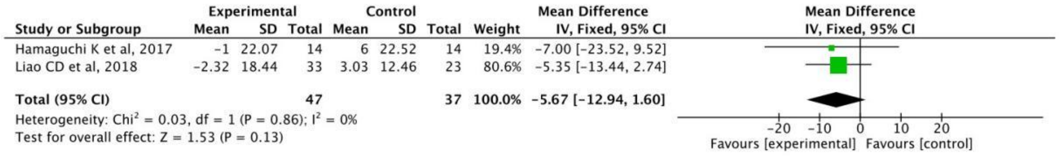

Fig.c. Forest plot of Quality of Life Outcomes

2.4. Hs-CRP

	Experimental			Control				Std. Mean Difference
Study or Subgroup	Mean	SD	Total	Mean	SD	Total	Weight	IV, Random, 95% CI
Huang SW et al, 2017	−0.2	3.03	18	0.5	2.88	17	29.3%	−0.23 [−0.90, 0.43]
Jung WS et al, 2024	−0.32	0.83	14	0.14	0.72	14	23.9%	−0.57 [−1.33, 0.18]
Kim H et al, 2016	0.1	1.91	35	−0.2	2	34	46.8%	0.15 [−0.32, 0.62]
**Total (95% CI)**			**67**			**65**	**100.0%**	**−0.13 [−0.55, 0.28]**

Heterogeneity: $Tau^2 = 0.04$; $Chi^2 = 2.74$, df = 2 (P = 0.25); $I^2 = 27\%$
Test for overall effect: Z = 0.63 (P = 0.53)

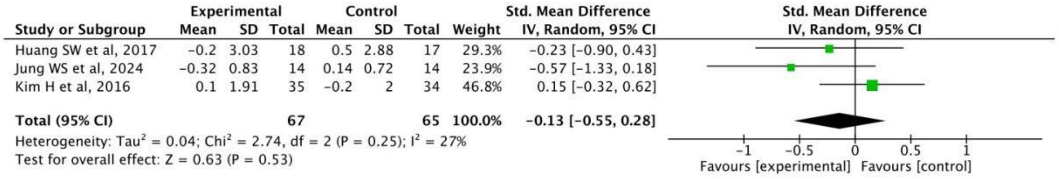

Fig.d. Forest plot of Hs-CRP

Appendix 3 Subgroup analysis
Result

3.1 Subgroup analyses-Type of exercise

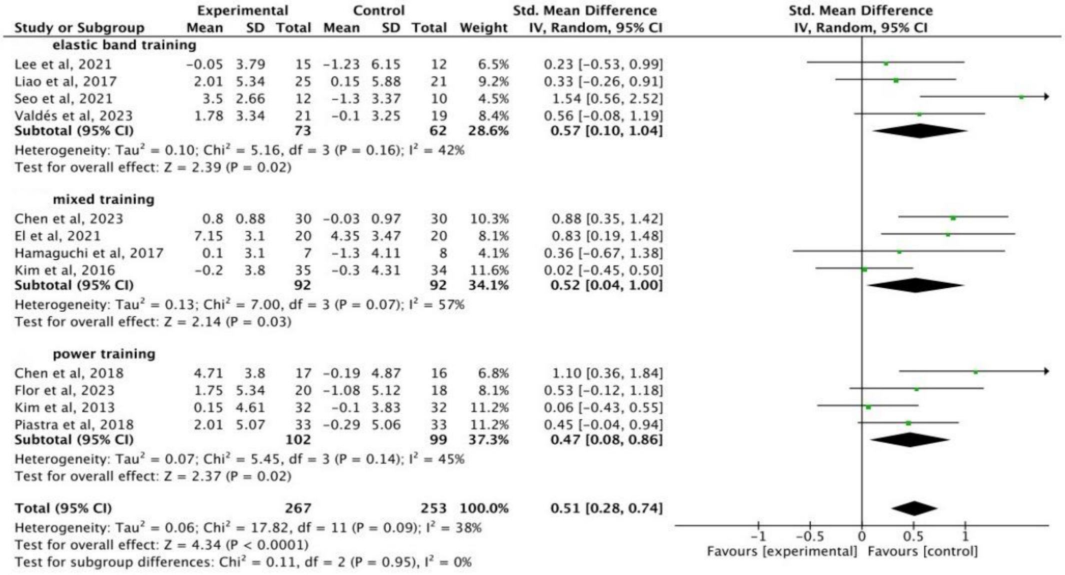

Fig.e. Forest plot of subgroup analysis of the effect of frequency on Grip Strength

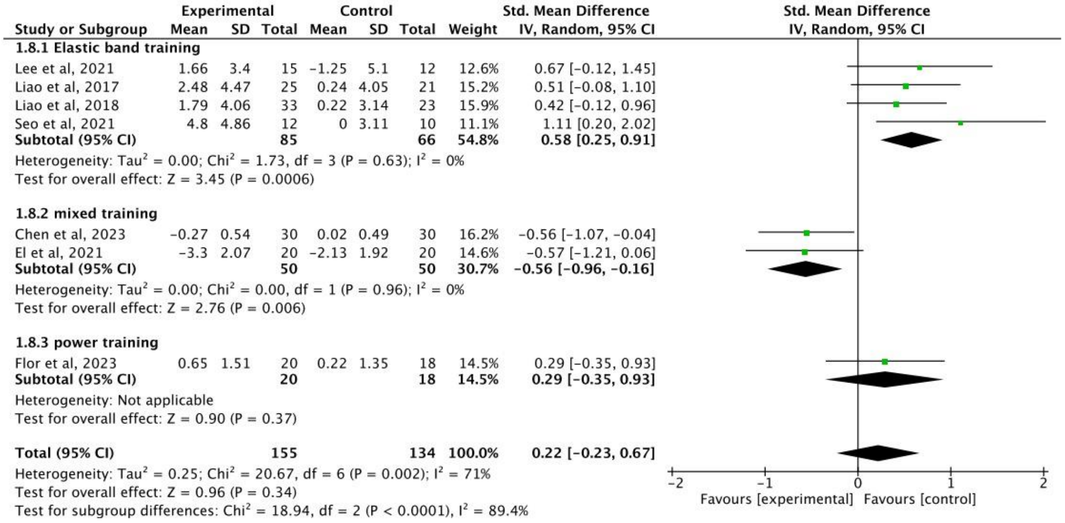

Fig.f. Forest plot of subgroup analysis of the effect of exercise on Timed Chair Rise

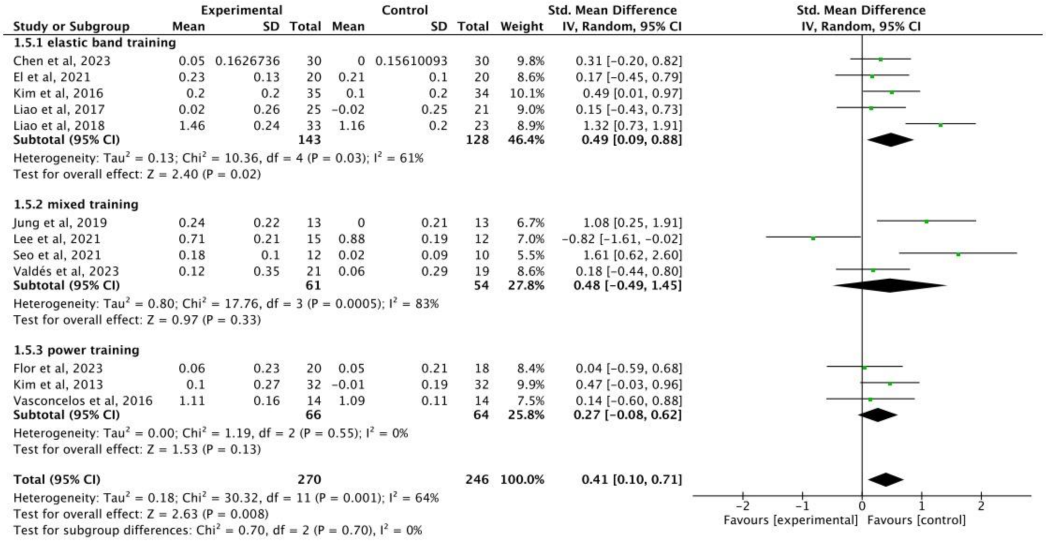

Fig.g. Forest plot of subgroup analysis of the effect of exercise on Gait Speed

3.2 Subgroup analyses - Frequency

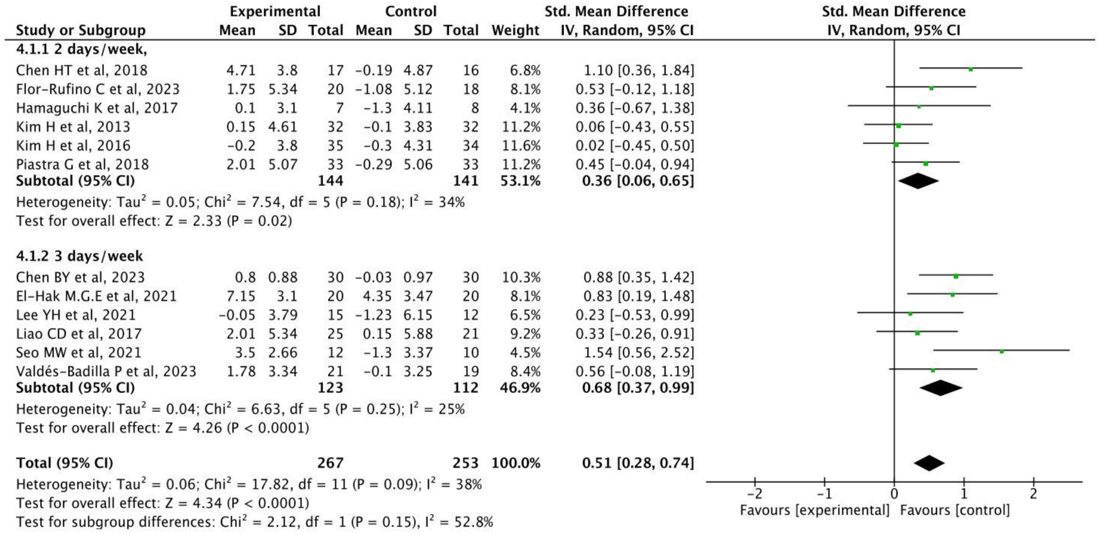

Fig.h. Forest plot of subgroup analysis of the effect of frequency on Grip Strength

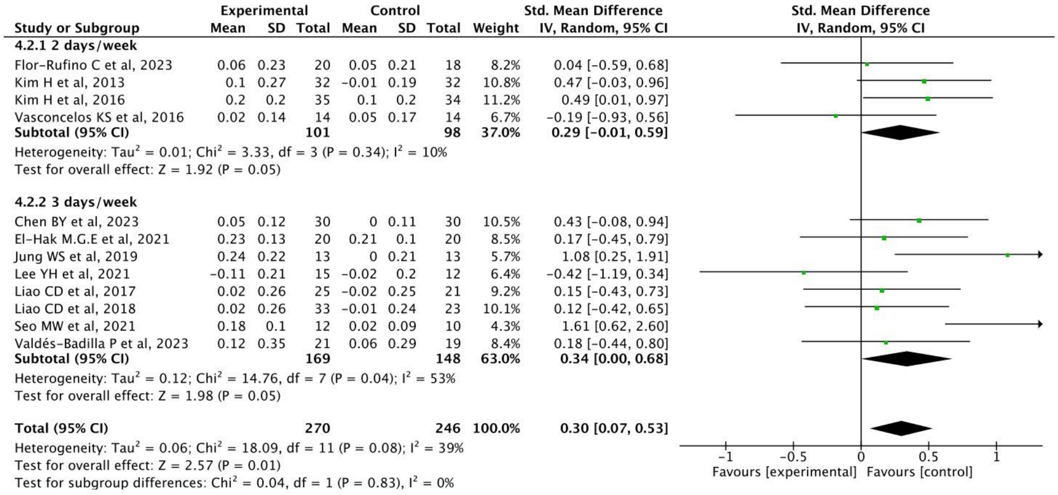

Fig.i. Forest plot of subgroup analysis of the effect of frequency on Gait Speed

3.3 Subgroup analyses Duration of time

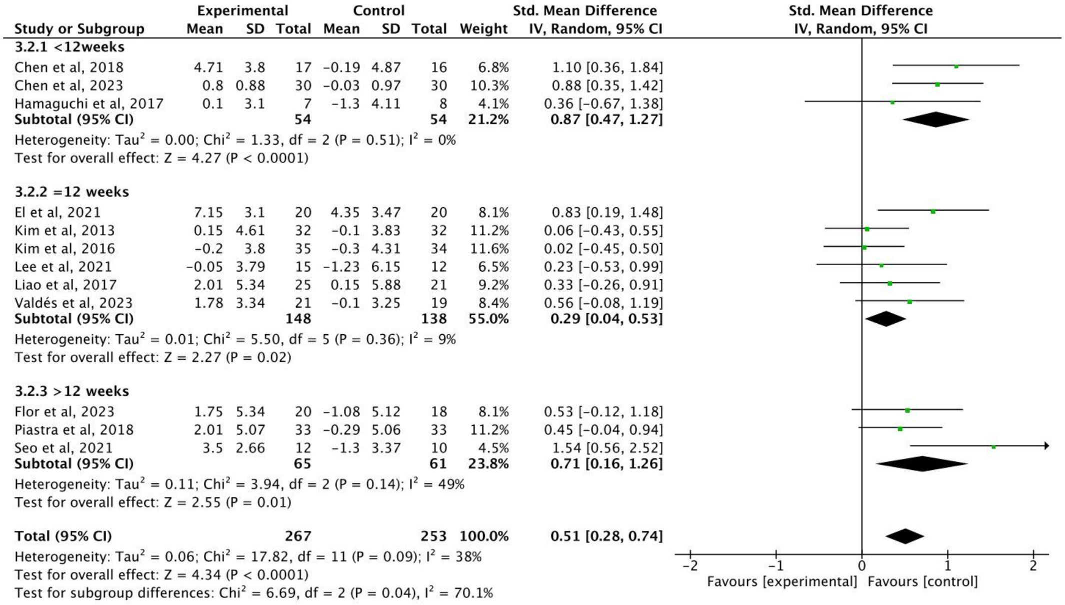

Fig.j. Forest plot of subgroup analysis of the effect of duration on Gaip Strength

Study or Subgroup	Experimental Mean	Experimental SD	Experimental Total	Control Mean	Control SD	Control Total	Weight	Std. Mean Difference IV, Random, 95% CI
**3.3.1 <12 weeks**								
Chen et al, 2023	0.05	0.12	30	0	0.11	30	10.5%	0.43 [−0.08, 0.94]
Vasconcelos et al, 2016	0.02	0.14	14	0.05	0.17	14	6.7%	−0.19 [−0.93, 0.56]
**Subtotal (95% CI)**			**44**			**44**	**17.2%**	**0.18 [−0.41, 0.77]**
Heterogeneity: Tau^2 = 0.08; Chi^2 = 1.79, df = 1 (P = 0.18); I^2 = 44%								
Test for overall effect: Z = 0.60 (P = 0.55)								
**3.3.2 =12 weeks**								
El et al, 2021	0.23	0.13	20	0.21	0.1	20	8.5%	0.17 [−0.45, 0.79]
Jung et al, 2019	0.24	0.22	13	0	0.21	13	5.7%	1.08 [0.25, 1.91]
Kim et al, 2013	0.1	0.27	32	−0.01	0.19	32	10.8%	0.47 [−0.03, 0.96]
Kim et al, 2016	0.2	0.2	35	0.1	0.2	34	11.2%	0.49 [0.01, 0.97]
Lee et al, 2021	−0.11	0.21	15	−0.02	0.2	12	6.4%	−0.42 [−1.19, 0.34]
Liao et al, 2017	0.02	0.26	25	−0.02	0.25	21	9.2%	0.15 [−0.43, 0.73]
Liao et al, 2018	0.02	0.26	33	−0.01	0.24	23	10.1%	0.12 [−0.42, 0.65]
Valdés et al, 2023	0.12	0.35	21	0.06	0.29	19	8.4%	0.18 [−0.44, 0.80]
**Subtotal (95% CI)**			**194**			**174**	**70.3%**	**0.28 [0.05, 0.52]**
Heterogeneity: Tau^2 = 0.02; Chi^2 = 8.83, df = 7 (P = 0.26); I^2 = 21%								
Test for overall effect: Z = 2.34 (P = 0.02)								
**3.3.3 >12 weeks**								
Flor et al, 2023	0.06	0.23	20	0.05	0.21	18	8.2%	0.04 [−0.59, 0.68]
Seo et al, 2021	0.18	0.1	12	0.02	0.09	10	4.3%	1.61 [0.62, 2.60]
**Subtotal (95% CI)**			**32**			**28**	**12.5%**	**0.78 [−0.75, 2.31]**
Heterogeneity: Tau^2 = 1.04; Chi^2 = 6.79, df = 1 (P = 0.009); I^2 = 85%								
Test for overall effect: Z = 1.00 (P = 0.32)								
**Total (95% CI)**			**270**			**246**	**100.0%**	**0.30 [0.07, 0.53]**
Heterogeneity: Tau^2 = 0.06; Chi^2 = 18.09, df = 11 (P = 0.08); I^2 = 39%								
Test for overall effect: Z = 2.57 (P = 0.01)								
Test for subgroup differences: Chi^2 = 0.51, df = 2 (P = 0.77), I^2 = 0%								

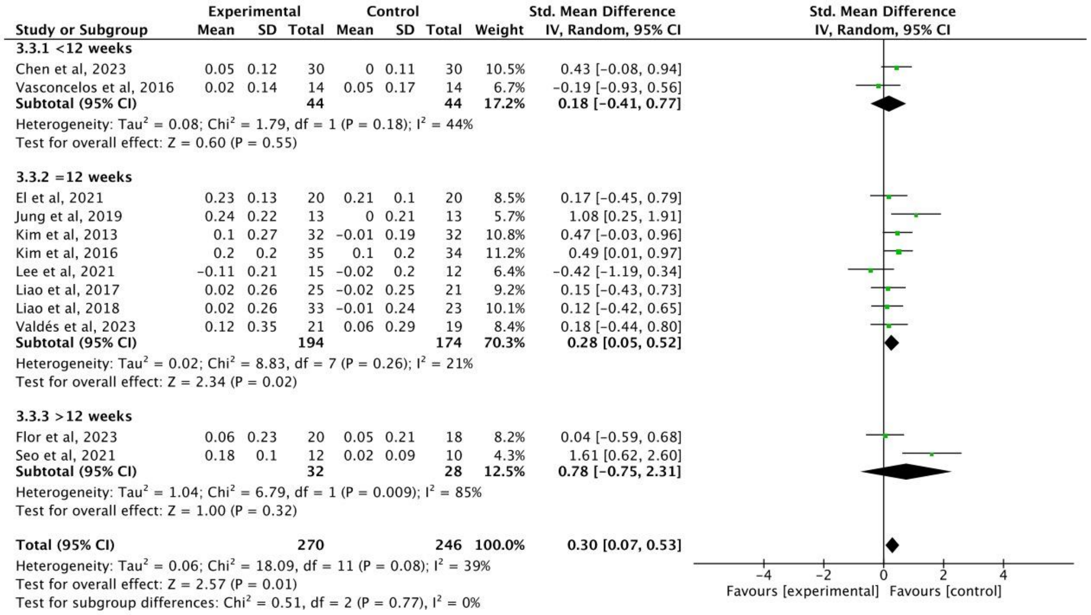

Fig.k. Forest plot of subgroup analysis of the effect of duration on Gait Speed

3.4 Subgroup analyses - Diagnosis

Study or Subgroup	Experimental Mean	Experimental SD	Experimental Total	Control Mean	Control SD	Control Total	Weight	Std. Mean Difference IV, Random, 95% CI
**7.8.1 EWGSOP**								
Chen BY et al, 2023	−0.27	0.54	30	0.02	0.49	30		Not estimable
El-Hak M.G.E et al, 2021	−3.3	2.07	20	−2.13	1.92	20	14.6%	−0.57 [−1.21, 0.06]
Flor-Rufino C et al, 2023	0.65	1.51	20	0.22	1.35	18	14.5%	0.29 [−0.35, 0.93]
Lee YH et al, 2021	1.66	3.4	15	−1.25	5.1	12	12.6%	0.67 [−0.12, 1.45]
Liao CD et al, 2017	2.48	4.47	25	0.24	4.05	21	15.2%	0.51 [−0.08, 1.10]
Seo MW et al, 2021	4.8	4.86	12	0	3.11	10	11.1%	1.11 [0.20, 2.02]
**Subtotal (95% CI)**			**92**			**81**	**68.0%**	**0.36 [−0.18, 0.89]**
Heterogeneity: Tau^2 = 0.24; Chi^2 = 11.67, df = 4 (P = 0.02); I^2 = 66%								
Test for overall effect: Z = 1.31 (P = 0.19)								
**7.8.2 AWGS**								
Chen BY et al, 2023	−0.27	0.54	30	0.02	0.49	30	16.2%	−0.56 [−1.07, −0.04]
**Subtotal (95% CI)**			**30**			**30**	**16.2%**	**−0.56 [−1.07, −0.04]**
Heterogeneity: Not applicable								
Test for overall effect: Z = 2.11 (P = 0.04)								
**7.8.3 Others**								
Liao CD et al, 2018	1.79	4.06	33	0.22	3.14	23	15.9%	0.42 [−0.12, 0.96]
**Subtotal (95% CI)**			**33**			**23**	**15.9%**	**0.42 [−0.12, 0.96]**
Heterogeneity: Not applicable								
Test for overall effect: Z = 1.52 (P = 0.13)								
**Total (95% CI)**			**155**			**134**	**100.0%**	**0.22 [−0.23, 0.67]**
Heterogeneity: Tau^2 = 0.25; Chi^2 = 20.67, df = 6 (P = 0.002); I^2 = 71%								
Test for overall effect: Z = 0.96 (P = 0.34)								
Test for subgroup differences: Chi^2 = 8.33, df = 2 (P = 0.02), I^2 = 76.0%								

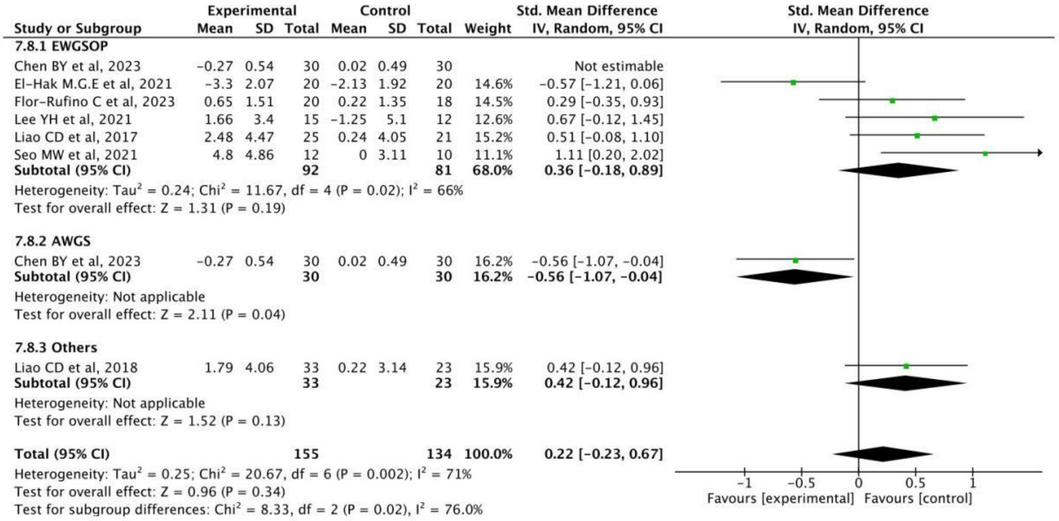

Fig.l. Forest plot of subgroup analysis of the effect of duration on Timed Chair Rise

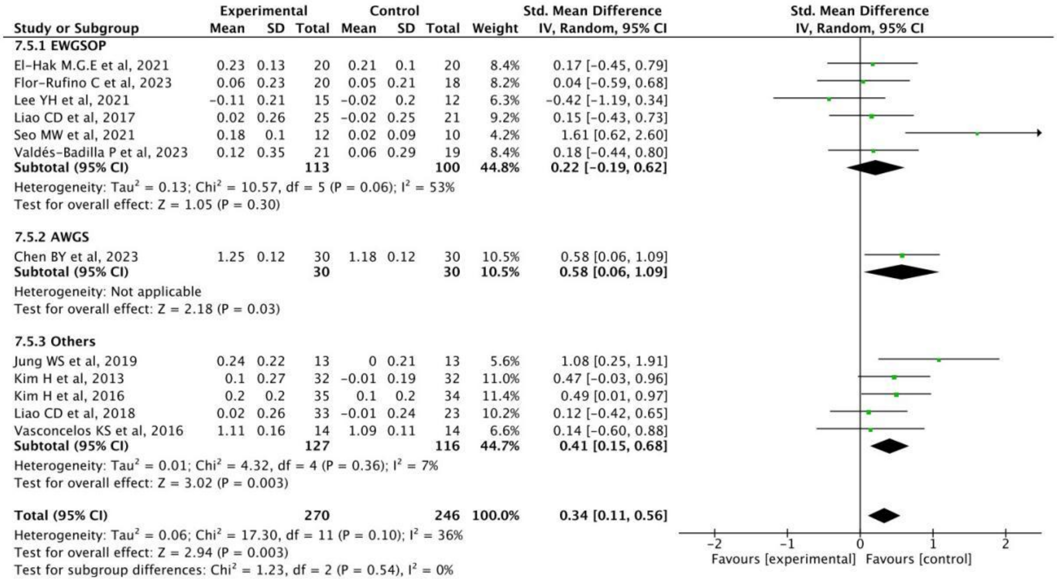

Fig.m. Forest plot of subgroup analysis of the effect of duration on Gait Speed

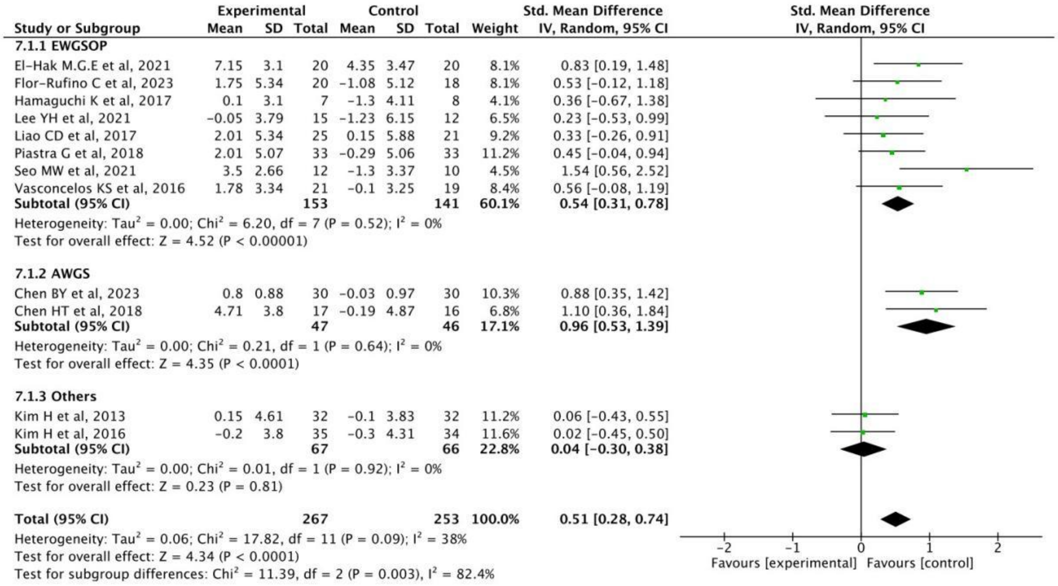

Fig.n. Forest plot of subgroup analysis of the effect of duration on Gaip Strength

Appendix 4 Funnel plot

Result

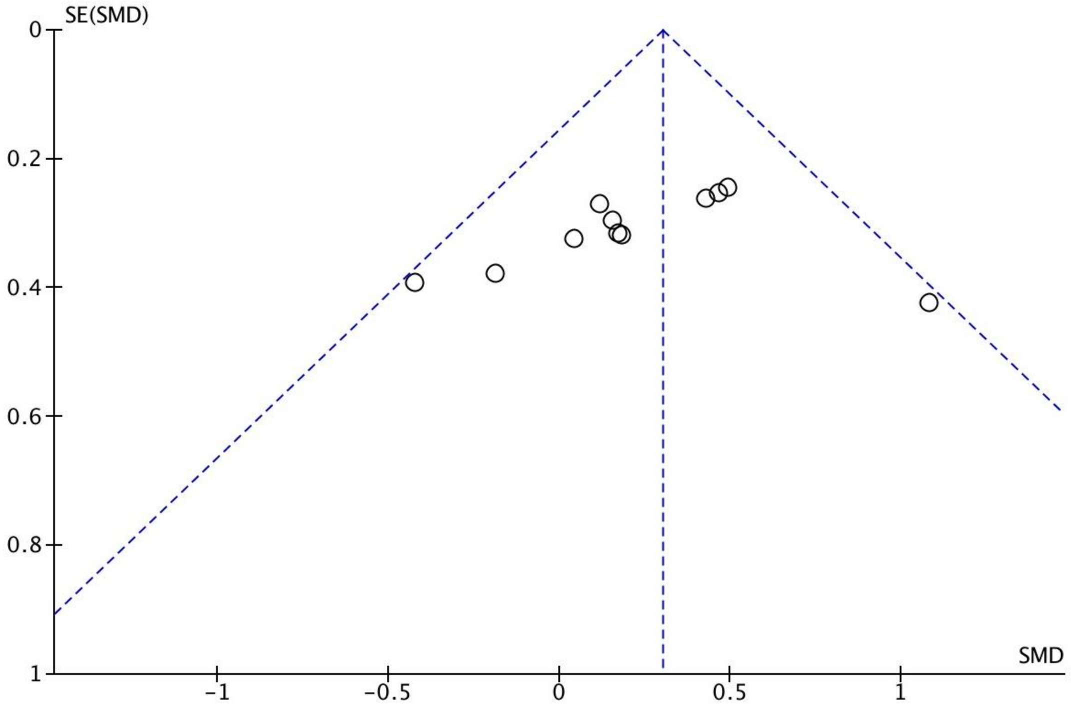

Fig.o. Funnel plot of grip strength

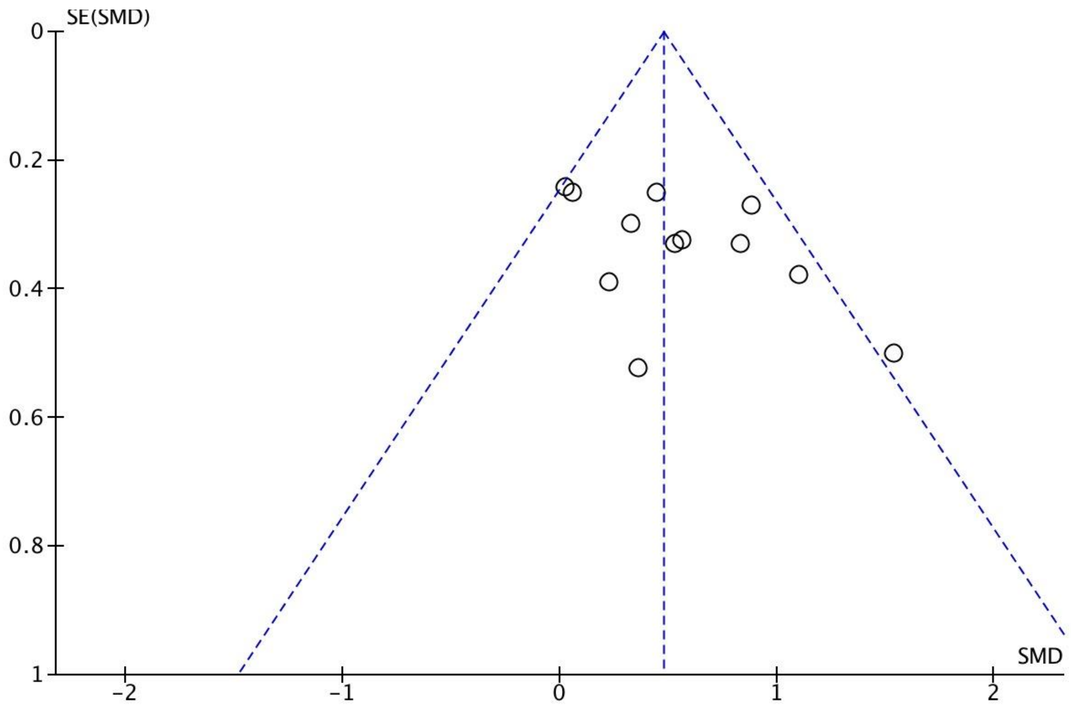

Fig.p. Funnel plot of gait speed

Appendix 4 PRISMA checklist

PRISMA checklist			
**Section and Topic**	**Item #**	**Checklist item**	**Location where item is reported**
**TITLE**			
Title	1	Identify the report as a systematic review.	Title
**ABSTRACT**			
Abstract	2	See the PRISMA 2020 for Abstracts checklist.	Supplementary
**INTRODUCTION**			
Rationale	3	Describe the rationale for the review in the context of existing knowledge.	Introduction
Objectives	4	Provide an explicit statement of the objective(s) or question(s) the review addresses.	Introduction
**METHODS**			
Eligibility criteria	5	Specify the inclusion and exclusion criteria for the review and how studies were grouped for the syntheses.	Method
Information sources	6	Specify all databases, registers, websites, organisations, reference lists and other sources searched or consulted to identify studies. Specify the date when each source was last searched or consulted.	Method
Search strategy	7	Present the full search strategies for all databases, registers and websites, including any filters and limits used.	Supplementary table
Selection process	8	Specify the methods used to decide whether a study met the inclusion criteria of the review, including how many reviewers screened each record and each report retrieved, whether they worked independently, and if applicable, details of automation tools used in the process.	Method
Data collection process	9	Specify the methods used to collect data from reports, including how many reviewers collected data from each report, whether they worked independently, any processes for obtaining or confirming data from study investigators, and if applicable, details of automation tools used in the process.	Method
Data items	10a	List and define all outcomes for which data were sought. Specify whether all results that were compatible with each outcome domain in each study were sought (e.g. for all measures, time points, analyses), and if not, the methods used to decide which results to collect.	Method
	10b	List and define all other variables for which data were sought	Method

		(e.g. participant and intervention characteristics, funding sources). Describe any assumptions made about any missing or unclear information.	
Study risk of bias assessment	11	Specify the methods used to assess risk of bias in the included studies, including details of the tool(s) used, how many reviewers assessed each study and whether they worked independently, and if applicable, details of automation tools used in the process.	Method
Effect measures	12	Specify for each outcome the effect measure(s) (e.g. risk ratio, mean difference) used in the synthesis or presentation of results.	Method
Synthesis methods	13a	Describe the processes used to decide which studies were eligible for each synthesis (e.g. tabulating the study intervention characteristics and comparing against the planned groups for each synthesis (item #5)).	Method
	13b	Describe any methods required to prepare the data for presentation or synthesis, such as handling of missing summary statistics, or data conversions.	Method
	13c	Describe any methods used to tabulate or visually display results of individual studies and syntheses.	Methods
	13d	Describe any methods used to synthesize results and provide a rationale for the choice(s). If meta-analysis was performed, describe the model(s), method(s) to identify the presence and extent of statistical heterogeneity, and software package(s) used.	Methods
	13e	Describe any methods used to explore possible causes of heterogeneity among study results (e.g. subgroup analysis, meta-regression).	Method
	13f	Describe any sensitivity analyses conducted to assess robustness of the synthesized results.	Method
Reporting bias assessment	14	Describe any methods used to assess risk of bias due to missing results in a synthesis (arising from reporting biases).	Method
Certainty assessment	15	Describe any methods used to assess certainty (or confidence) in the body of evidence for an outcome.	Method
**RESULTS**			
Study selection	16a	Describe the results of the search and selection process, from the number of records identified in the search to the number of studies included in the review, ideally using a flow diagram.	Results
	16b	Cite studies that might appear to meet the inclusion criteria, but which were excluded, and explain why they were excluded.	Results

Study characteristics	17	Cite each included study and present its characteristics.	Results
Risk of bias in studies	18	Present assessments of risk of bias for each included study.	Resultss
Results of individual studies	19	For all outcomes, present, for each study: (a) summary statistics for each group (where appropriate) and (b) an effect estimate and its precision (e.g. confidence/credible interval), ideally using structured tables or plots.	Results
Results of syntheses	20a	For each synthesis, briefly summarise the characteristics and risk of bias among contributing studies.	Results
	20b	Present results of all statistical syntheses conducted. If meta-analysis was done, present for each the summary estimate and its precision (e.g. confidence/credible interval) and measures of statistical heterogeneity. If comparing groups, describe the direction of the effect.	Results
	20c	Present results of all investigations of possible causes of heterogeneity among study results.	Results
	20d	Present results of all sensitivity analyses conducted to assess the robustness of the synthesized results.	Results
Reporting biases	21	Present assessments of risk of bias due to missing results (arising from reporting biases) for each synthesis assessed.	Results
Certainty of evidence	22	Present assessments of certainty (or confidence) in the body of evidence for each outcome assessed.	Results
**DISCUSSION**			
Discussion	23a	Provide a general interpretation of the results in the context of other evidence.	Discussion
	23b	Discuss any limitations of the evidence included in the review.	Discussion
	23c	Discuss any limitations of the review processes used.	Discussion
	23d	Discuss implications of the results for practice, policy, and future research.	Discussion
**OTHER INFORMATION**			
Registration and protocol	24a	Provide registration information for the review, including register name and registration number, or state that the review was not registered.	registration number CRD42024608200
	24b	Indicate where the review protocol can be accessed, or state that a protocol was not prepared.	Not prepared
	24c	Describe and explain any amendments to information provided at registration or in the protocol.	Not prepared
Support	25	Describe sources of financial or non-financial support for the	Funding information

		review, and the role of the funders or sponsors in the review.	
Competing interests	26	Declare any competing interests of review authors.	Declaration of Competing Interest
Availability of data, code and other materials	27	Report which of the following are publicly available and where they can be found: template data collection forms; data extracted from included studies; data used for all analyses; analytic code; any other materials used in the review.	Yes

Appendix 6 Detailed Description of Interventions

Result

Intervention type	Intervention content
**Elastic band resistance training**	Progressive resistance exercises using elastic bands targeting major muscle groups, including upper limbs, lower limbs, and trunk muscles. Resistance levels were gradually increased based on participants' tolerance.
**Circuit training**	Walking in place, shoulder presses and squats, twist dashes, lunges, jumping jacks, kickbacks, modified push–ups, crunches, hip bridges, bird dogs;
**Core trainings**	Drawing–in maneuver, Diaphragmatic breathing exercise, Pelvic bridging exercise, Abdominal crunches, Abdominal "tuck in" in quadruped position, Cat–camel motions, Bird–dog exercise
**Kettlebell training**	kettlebell swing, kettlebell deadlift, kettlebell goblet squat, squat lunge, kettlebell row, single arm kettlebell row, biceps curl, triceps extension, two–arm kettlebell military press, Turkish get up, and comprehensive dynamic workout;
**Low-load muscle strengthening**	standing/on the ground 30′ muscle toning at low/moderate intensity for different muscular districts (primarily abdominal and both lower and upper limbs) with low weight loads (0.5, 1, or 1.5 Kg)
**Stretching, Strengthening, Balance and Gait Training**	A combined program including flexibility exercises (static stretching of major muscle groups), muscle strengthening (bodyweight or light resistance), balance training (single-leg stance), and gait training (walking practice and coordination exercises).
**Tai Chi and progressive resistance training**	Moderate-intensity multicomponent program including 24-form Tai Chi and progressive elastic band resistance exercises targeting upper and lower body muscle groups
**High-Intensity Circuit Resistance Training (HIRT)**	A high-intensity resistance training protocol performed in a circuit format, involving short bursts of vigorous exercises with limited recovery periods.
**Open and closed chain training**	open and closed kinetic chain exercises targeting lower limb strength, endurance, and power, with progressive transition to high-velocity movements
